# Overall reductions in functional brain activation are associated with falls in older adults: an fMRI study

**DOI:** 10.3389/fnagi.2013.00091

**Published:** 2013-12-19

**Authors:** Lindsay S. Nagamatsu, Lara A. Boyd, Chun Liang Hsu, Todd C. Handy, Teresa Liu-Ambrose

**Affiliations:** ^1^Attentional Neuroscience Laboratory, Department of Psychology, University of British ColumbiaVancouver, BC, Canada; ^2^Brain Behaviour Laboratory, Department of Physical Therapy, University of British ColumbiaVancouver, BC, Canada; ^3^Brain Research Centre, Centre for Brain Health, University of British ColumbiaVancouver, BC, Canada; ^4^Aging, Mobility, and Cognitive Neuroscience Laboratory, Department of Physical Therapy, University of British ColumbiaVancouver, BC, Canada

**Keywords:** falls, older adults, fMRI, executive cognitive functions, Flanker task, medial frontal gyrus

## Abstract

Falls are a common geriatric condition, and while impaired cognitive function has been identified as a key risk factor, the neural correlates that contribute to reduced executive functioning and falls currently remain unknown. In this study, community-dwelling adults aged 65–75 years were divided into two groups based on their recent history of falls (fallers versus non-fallers). All participants completed the Flanker task during functional magnetic resonance imaging (fMRI). We examined the hemodynamic response of congruent and incongruent trials separately in order to separate the relative contribution of each trial type as a function of falls history. We found that fallers exhibited a smaller difference in functional activation between congruent and incongruent trials relative to non-fallers, as well as an overall reduction in level of blood-oxygen-level dependent response. Of particular note, the medial frontal gyrus – a region implicated in motor planning – demonstrated hypo-activation in fallers, providing evidence that the prefrontal cortex might play a central role in falls risk in older adults.

## INTRODUCTION

Falls are a common geriatric syndrome, with approximately 30% of community dwelling seniors experiencing one or more per year. Experts consider falls to be non-random events (Grimley-Evans, 1990) and reliable risk factors for falls have been identified. Broadly, falls risk factors can be divided into two distinct categories: (1) physical risk factors, or (2) cognitive risk factors. Physical risk factors include mobility and balance issues, which can be assessed using multiple physiological components (Lord et al., 2003).

Cognitive dysfunction is also a key falls risk factor (Tinetti et al., 1988). Within the multiple domains of cognitive function, impaired executive functioning – the ability to concentrate, to attend selectively, to plan and strategize – is associated with falls (Lundin-Olsson et al., 1997; Rapport et al., 1998; Anstey et al., 2006). More specifically, the executive cognitive processes of response inhibition, as measured by the Stroop Color-Word test (Lord and Fitzpatrick, 2001; Liu-Ambrose et al., 2008a), and set-shifting, as measured by the Trail Making test (Corrigan and Hinkeldey, 1987), appear to be most relevant to falls.

It is well-known that both physical and cognitive abilities have neural concomitants, suggesting that altered functional brain responses may play a prominent role in falls risk. This hypothesis is substantiated by recent neuroimaging evidence demonstrating neural differences between fallers and non-fallers. Specifically, we previously demonstrated that the posterior lobe of the right cerebellum had significantly reduced hemodynamic response among seniors with a recent history of falls compared with non-fallers while performing the Eriksen Flanker task – a task requiring selective attention and conflict resolution – as assessed by fMRI (Liu-Ambrose et al., 2008b). Additionally, in a study examining the relationship between functional activation and physiological falls risk, we found significant associations between the left frontal orbital cortex extending towards the insula, and the paracingulate gyrus extending towards the anterior cingulate gyrus with change in physiological falls risk over a 12-month period (Nagamatsu et al., 2011a). Taken together, these studies provide evidence that altered brain function may be associated with falls.

In our previous study (Liu-Ambrose et al., 2008b), we compared functional activation on the Flanker task between fallers and non-fallers as the difference between incongruent and congruent trials. In other words, we examined areas that showed greater activation during incongruent trials – the more cognitively difficult condition – using congruent trials as a baseline. Given our previous methodology, it remains unclear whether there are overall differences in level of blood-oxygen-level dependent (BOLD) signal in fallers relative to non-fallers, as well as the independent contribution of each type of condition (congruent and incongruent) to the results we obtained. For example, was our observed difference due to lower activation of incongruent trials or higher activation of congruent trials in fallers? This is currently unknown because we previously only examined the difference score between conditions, rather than directly comparing percent signal change between groups. Of relevance for our current study, the two different trial types engage separate cognitive processes. In particular, incongruent trials require the engagement of selective attention and response inhibition; notably, these are key executive functions that have been tied to falls (Lord and Fitzpatrick, 2001; Holtzer et al., 2007). Therefore, examining differences in activation within each condition separately could provide valuable information regarding the specific neural correlates that contribute to cognitive alterations in fallers.

The main purpose of this 2-month prospective observational study was to examine the independent contributions of congruent and incongruent trial types on the hemodynamic response in older women with and without a recent history of falls. We hypothesized that we would observe differences in the BOLD response between fallers and non-fallers as a function of trial type. Such results would have the potential to inform us about the specific underlying neural differences that may contribute to impaired executive cognitive processing – and falls – in older adults.

## MATERIALS AND METHODS

### PARTICIPANTS

Our sample consisted of a subset of 83 women out of 158 participants in a 12-month randomized controlled trial (RCT) of resistance training with three experimental groups: balance and tone exercises, once weekly resistance training, and twice weekly resistance training (Liu-Ambrose et al., 2010). The subset was selected based on MRI scanning eligibility and consent. Participants in the RCT included community-dwelling women who: (1) were aged 65–75 years; (2) obtained a score ≥24 on the Mini Mental Status Examination (MMSE); and (3) had a visual acuity of at least 20/40, with or without corrective lenses. Participants were excluded if they: (1) had a diagnosed neurodegenerative disease and/or stroke; (2) were taking psychotropic drugs; (3) did not speak and understand English; (4) had moderate to significant impairment with activities of daily living, as determined by interview; (5) were taking cholinesterase inhibitors within the last 12 months; (6) were taking anti-depressants within the last 6 months; or (7) were on estrogen replacement therapy within the last 12 months. Data reported here are from the baseline assessments of the trial. This study was approved by the Vancouver Coastal Health Research Institute and the University of British Columbia’s Clinical Research Ethics Board. All participants provided written informed consent.

### TWO-MONTH OBSERVATION PERIOD

Falls were assessed during a 2-month observation period. During the observation period, participants completed baseline assessments, were randomly allocated into their exercise groups, and underwent an orientation for training. Falls were defined as “unintentionally coming to the ground or some lower level and other than as a consequence of sustaining a violent blow, loss of consciousness, sudden onset of paralysis as in stroke or an epileptic seizure” (Kellogg International Work Group, 1987). Falls were recorded within the 2-month observation period using calendars that were completed daily and submitted monthly. Participants were instructed to record falls or absence of falls daily on the calendar; they were instructed that if they fell, they were to phone the research coordinator at their earliest convenience. The research coordinator noted the details of all falls reported, such as the specific circumstances and whether or not any injuries were sustained.

### DESCRIPTIVE VARIABLES

Global cognitive state was assessed using the MMSE (Folstein et al., 1975). General health and socioeconomic status were ascertained by a questionnaire. Participants completed a 15-minute physician assessment to confirm current health status and eligibility for study. History of falls in the last 12 months was reported during an interview with the study physician.

General mobility was assessed by the Timed Up and Go (TUG) Test (Podsiadlo and Richardson, 1991). Participants were instructed to rise from a standard chair with arms, walk a distance of three meters, turn, walk back to the chair, and sit down again. The mean of two trials was calculated and used for statistical analysis. Physiological falls risk profile was assessed by the short form of the Physiological Profile Assessment (PPA; Prince of Wales Medical Research Institute, Randwick, Sydney, NSW, Australia). The PPA is a valid and reliable measure of falls risk in older people (Lord et al., 2003). Based on a participant’s performance of five physiological domains (postural sway, reaction time, strength, proprioception, and vision), the PPA computes a global falls risk score (standardized score) that has a 75% predictive accuracy for falls in older people. Global PPA scores below 0 indicate a low risk of falling, scores between 0 and 1 indicate a mild risk of falling, scores between 1 and 2 indicate a moderate risk of falling, and scores above 2 indicate a high risk of falling.

### PRIMARY OUTCOME MEASURE: FUNCTIONAL MAGNETIC RESONANCE IMAGING

Brain activation was examined using functional magnetic resonance imaging (fMRI). Functional MRI is a non-invasive neuroimaging technique, which measures the BOLD signal in the brain, enabling us to make inferences regarding regional activation during performance of a cognitive task. We collected baseline fMRI data from all 83 participants with a 3.0T Intera Achieva MRI scanner (Philips Medical Systems Canada, Markham, ON, Canada) in the UBC High Field MRI Centre at the UBC Hospital.

#### Scanning parameters

Transverse echo-planar imaging (EPI) images in-plane with the AC-PC line were acquired using a gradient-echo pulse sequence and sequential slice acquisition (TR = 2000 ms, TE = 30 ms, flip angle = 90°, 36 contiguous slices at 3 mm skip 1 mm, in-plane resolution of 128 × 128 pixels reconstructed in a FOV of 240 mm). Each functional run began with four TR’s during which no data were acquired to allow for steady-state tissue magnetization. A total of 148 EPI volumes were collected in each functional run, and six functional runs were performed by each participant. High-resolution, T1-weighted axial images were also collected for each participant to allow anatomical and functional images to be co-registered during data analysis (TR = 8 ms, TE = 3.7 ms, bandwidth = 2.26 kHz, voxel size = 1 × 1 × 1 mm).

#### Cognitive task parameters

**FIGURE 1:**
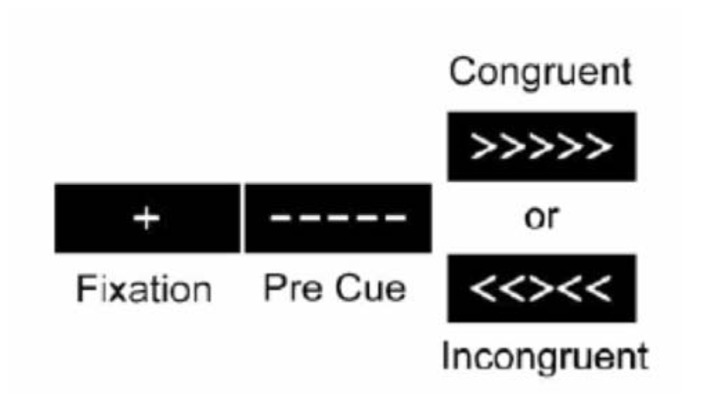
**The Eriksen Flanker task that participants completed in during fMRI scanning.** Participants were required to indicate the direction of the central arrow (left versus right) while ignoring flanking peripheral arrows as quickly and accurately as possible.

The experiment was a slow event-related design. During scanning, participants performed a modified Ericksen Flanker task (**Figure 1**). On each trial participants viewed a visual display that contained five arrows and were required to indicate the direction the central arrow was pointing (left versus right) while ignoring the two flanking arrows on each side. On half the trials, the flanking arrows pointed in the same direction as the central arrow cue (e.g., <<<<<; congruent condition), and on the other half of the trials the flanking arrows pointed in the opposite direction (e.g., >><>>; incongruent condition). The primary measure of executive cognitive functioning for the Erikson Flanker task stems from comparing behavioral and neural responses between incongruent and congruent trials; the former condition requires engaging both selective attention and response inhibition in order to ignore the flanking distracters that engender a response incompatible with the required target response. Importantly, this paradigm is sensitive to age-related decrements in attentional control (Kramer et al., 1999).

During fMRI scanning, participants viewed stimuli projected onto a screen on the back wall behind the scanner that could be seen via a mirror attached to the head coil. A central fixation cross was presented for 500 ms at the beginning of each trial. The target stimuli (arrows) were then shown for 2000 ms. An average of 13500 ms of blank screen was presented between each trial, jittered between 11500 and 15000 ms. Participants indicated the direction of the arrows via button presses. Reaction time was recorded in milliseconds. Each participant underwent six successive 5-minute blocks where they were presented with 17 trials that were first-order counter-balanced, such that congruent and incongruent trials followed each other equally.

#### fMRI processing and analysis

Functional images were generated using Analysis of Functional NeuroImages (AFNI) software (Cox, 1996). Time-series images were spatially registered in three dimensions to minimize effects of head motion. Runs with average motion exceeding 4 mm of translation or 3° of rotation were excluded (Kimberley et al., 2008). A deconvolution analysis was used to generate impulse response functions (IRFs) of the fMRI signal on a voxel-wise basis. This analysis produced an estimate of the haemodynamic response for main effect (i.e., congruent, incongruent) relative to a baseline state (rest) for the first eight TRs without making *a priori* assumptions about the shape, delay, or magnitude of the IRF. Anatomical and functional images were then interpolated to volumes with 1 mm^3^ voxels, co-registered, converted to Talairach stereotaxic coordinate space, and blurred using a 4 mm Gaussian full-width half-maximum filter to compensate for inter-subject variability in anatomic and functional anatomy. Data were converted to percent signal change by time of repetition (TR). Activation foci were delineated using the Talairach atlas for the cerebral cortex (Talaraich and Tournoux, 1988) and the Schmahmann atlas for the cerebellum (Schmahmann et al., 1999, 2000).

#### Statistical analyzes

The primary behavioral outcome was interference on the Flanker task, which reflects reaction time on incongruent trials unbiased by differences in base reaction time. Interference was computed as the percent increase in reaction time to incongruent stimuli, over and above the average reaction time to congruent stimuli [(incongruent reaction time – congruent reaction time)/congruent reaction time] × 100 (Colcombe et al., 2004). Only correct responses are included in analysis.

For our fMRI data, we conducted a mixed-model analysis of variance (ANOVA). Specifically, to illustrate the overall effect of our experimental manipulations we tested the Group (fallers versus non-fallers) by Condition (congruent versus incongruent) interaction. To reduce the likelihood of false positives, the threshold for statistical significance was set at a *p* value of 0.01 (critical *F* = 9.05) and a minimum cluster size of 200 contiguous voxels was employed (Forman et al., 1995).

## RESULTS

### PARTICIPANTS

During the 2-month observation period, 14 out of 83 participants fell, resulting in a total of 15 falls. One of the 15 falls resulted in a hip fracture; eight caused moderate injuries (e.g., sprains and bruises). To create equal group sizes for statistical purposes, 14 of the remaining non-fallers were randomly selected using a random number generator in Microsoft Office (Excel) to comprise the “non-fallers” group. Demographic information for our participants is provided in **Table 1**. Fallers and non-fallers did not significantly differ on age, MMSE score, history of falls over the past 12 months, or TUG Test (all *p*’s > 0.10). There was a trend towards higher physiological falls risk for fallers compared with non-fallers, *t*(26) = -1.96, *p* = 0.06.

**Table 1:** Descriptive measures.

Variable	Fallers *n* = 14 Mean (SD)	Non-fallers *n* = 14 Mean (SD)
Age (year)	69.36 (2.95)	68.86 (2.96)
MMSE Score (max. 30 pts)	29.00 (0.88)	28.43 (0.94)
**Education (No, %)**
Less than Grade 9	0 (0.0)	0 (0.0)
Grades 9–12 without Certificate or Diploma	2 (14.3)	0 (0.0)
High School Certificate or Diploma	1 (7.1)	2 (14.3)
Trades or Professional Certificate or Diploma	1 (7.1)	2 (14.3)
University Certificate or Diploma	1 (7.1)	4 (28.6)
University Degree	9 (64.3)	6 (42.9)
Number of Falls in the past 12 months	0.71 (1.07)	0.29 (0.47)
Timed Up and Go Test (s)	6.70 (1.38)	6.14 (0.74)
PPA Score	0.58 (0.97)	0.08 (0.78)

### BEHAVIOR

Behavioral performance on the Flanker task was not significantly different between fallers and non-fallers (*p* = 0.17). However, there was a trend towards fallers performing better (i.e., less interference) than non-fallers (mean interference = 18.16, SD = 10.87 and 26.30, SD = 18.47 for fallers and non-fallers, respectively), which is consistent with our previous study (Liu-Ambrose et al., 2008b). Overall, accuracy was very high on the Flanker task (mean accuracy = 97.50%, SD = 6.11). There were no significant between-group differences in task accuracy, *p* = 0.33.

### FUNCTIONAL MAGNETIC RESONANCE IMAGING

**FIGURE 2:**
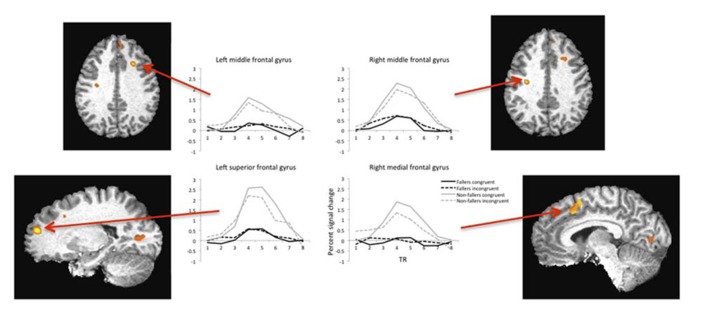
**Example hemodynamic responses during fMRI for four regions of interest plotted as a function of condition type (congruent versus incongruent) and group (fallers versus non-fallers).** Each region listed showed significantly higher activity in the non-fallers for the congruent condition. Note: images are shown in radiologic space (L = R).

Brain regions with significant activation as identified by a significant Group by Condition interaction (*F* = 9.05, *p* = 0.01) and their corresponding percent signal changes are shown in **Table 2**. There were 15 significantly active regions, including bilateral middle and superior frontal gyri, left inferior gyrus, and right superior temporal gyrus. To examine the pattern of results underlying each of the listed 15 interactions, we extracted the percent signal change of the BOLD response separately as a function of Group and Condition. Based on the percent signal change for the two trial types, it is evident that non-fallers exhibited larger differences in percent signal change *between* the two condition types (i.e., congruent and incongruent) compared with fallers (see **Figure 2**). Furthermore, the data reveal that overall non-fallers show a greater hemodynamic response in each of our 15 regions of interest (ROIs) relative to fallers.

**Table 2:** Regions of interest and percent signal change for the significant group by condition interaction.

Hemisphere	Area	Talaraich coordinates			Brodmann area	Fallers		Non-fallers	
		*X*	*Y*	*Z*		Congruent	Incongruent	Congruent	Incongruent
R	Medial frontal gyrus	5.9	63.5	14.1	10	0.1155	0.0706	1.8630	1.3347
L	Lentiform nucleus (putamen)	–25.2	–16.3	14.1		0.3583	0.3498	1.0807	1.1731
R	Cingulate gyrus	17.6	16.5	30.9	32	0.1711	–0.3741	0.8983	0.9637
L	Inferior frontal gyrus	–43.6	16.7	–10.2	47	0.1623	0.2403	0.2746	0.3873
R	Middle frontal gyrus	32.4	38.5	39.2	8	0.6856	0.7138	2.2816	1.9767
L	Middle frontal gyrus	–38.6	65.9	16.5	10	0.3473	0.4817	1.1410	1.0090
L	Subcallosal gyrus	–2.7	18.0	–14.4	25	0.4460	0.3236	2.1828	1.7486
L	Middle frontal gyrus	–51.1	14.7	29.0	9	0.3503	0.2264	1.5866	1.3446
L	Middle frontal gyrus	–21.9	–1.8	59.9	6	0.3969	0.3815	1.2027	1.3181
L	Parahippocampal gyrus	–40.1	–40.7	–0.1	19	–0.4926	–0.4822	0.5850	0.2696
R	Medial frontal gyrus	4.2	–11.8	53.9	6	0.7175	1.0006	2.3380	2.0153
R	Superior temporal gyrus	44.8	–39.6	12.8	41	–0.3750	–0.2965	0.5511	0.2110
L	Superior frontal gyrus	–21.8	59.4	–13.1	11	0.5451	0.5765	2.5699	2.1962
R	Superior frontal gyrus	13.3	43.2	44.7	8	0.1350	0.1018	0.7845	0.3352
L	Posterior cingulate	–17.8	–42.3	9.5	29	0.0456	0.0197	–0.0784	-0.2366

## DISCUSSION

Our study aimed to examine differences in hemodynamic response between senior fallers and non-fallers as a function of condition type during the Flanker task. In this regard, we report two key findings. First, fallers had a smaller difference in functional activation between congruent and incongruent trials relative to non-fallers. Second, fallers overall had a reduced level of functional activation compared with non-fallers. Taken together, our results align with the current prevailing notion that executive cognitive functions play a critical role in falls risk in older adults and bring to light several noteworthy points of discussion.

First, our results are consistent with previous reports that fallers have superior performance on the Flanker task relative to non-fallers (Liu-Ambrose et al., 2008b). While behavioral performance on the Flanker task in our current study was not significantly different between fallers and non-fallers, fallers did manifest a behavioral response pattern that was indicative of less interference from the flanking distractors, compared with the non-fallers. This has been interpreted as an overly narrow focus of spatial attention in fallers. Briefly put, studies in healthy young adults have demonstrated not only that the size of the attentional spotlight is flexible (Eriksen and St James, 1986), but also that the spotlight becomes more narrow and focused under conditions of high perceptual load (Handy and Mangun, 2000; Handy et al., 2001). Thus, within this context, our behavioral results suggest that fallers may have reduced attentional sensitivity in parafoveal vision and that this spotlight narrowing may be associated with deficits in visual-perceptual processing. This theory is corroborated by our neuroimaging results showing a negligible difference in percent signal change between incongruent and congruent trials in fallers. Specifically, that the stimuli are being processed similarly in the two conditions suggests that fallers may not be cognitively evaluating the peripheral arrows to the same extent as non-fallers.

Second, what might account for the overall reduction in the BOLD signal in fallers compared with non-fallers? This pattern of results could be the consequence of decreased neural function and/or structural integrity of the brain. In particular, neural changes inherent to pathological aging can alter neurovascular coupling, which is responsible for the BOLD response (D’Esposito et al., 2003). Functional activation has also been shown to be more distributed throughout the brain as a compensatory response to combat reduced processing capacity at local brain regions in order to maintain task performance (Cabeza, 2002). While this possibility is speculative at this point, changes in the distribution of activation between fallers and non-fallers is plausible; indeed, the idea that fallers may exhibit altered within- and between-network connectivity is something that is currently being examined (Hsu et al., in submission).

An alternative explanation for our results is that fallers might not have been paying as much attention to the task overall compared to non-fallers – thus resulting in an overall lower BOLD response. Indeed, previous research has pointed towards an association between attentional deficits and falls (Nagamatsu et al., 2009, 2013). We highlight, however, that we did not observe between-group differences on task accuracy during the Flanker task. Given that fallers demonstrated equivalent performance to non-fallers suggests that their level of attention was similar; therefore, our current data does not allude to the notion that a lack of attention in fallers is contributing to the reduced BOLD response. Additional research is necessary to further investigate the difference in BOLD response between fallers and non-fallers.

Finally, one specific region that we found to exhibit reduced activation in fallers compared with non-fallers – the right medial frontal gyrus (BA 10) – is of particular interest, due to its identified role as a “gateway,” functioning as a higher-level control mechanism to facilitate attentional orienting towards stimulus-related versus stimulus-independent thoughts (Burgess et al., 2007a,b; Smallwood et al., 2012). In particular, BA 10 has been implicated in self-generated thoughts pertinent for planning, reasoning, and engaging working memory (Christoff et al., 2003). Furthermore, a study by Boyd et al. (2009), found that activation in BA 10 was critical for planning goal-directed movements during a manual task. Importantly, such functions are essential for safe mobility and corroborate past evidence that older adults at-risk for falls demonstrate poor decision-making and judgments when crossing a stimulated virtual street (Nagamatsu et al., 2011b). Hence, future work should focus on examining the specific role of BA 10 and motor planning in fallers.

We acknowledge the limitations of our study. Our sample size is small and limited to women aged 65–75 years. Hence, future studies examining the relationship between falls and executive cognitive functioning should investigate whether such effects extend to a larger and more heterogeneous sample. Lastly, the cross-sectional nature of our study limits us to conclusions regarding the correlational associations between functional brain activation and falls status, rather than providing evidence for causal relationships. Future work examining the distribution of activation in the brain during an executive cognitive task in fallers is warranted.

In conclusion, our study is the first to provide evidence that older women with a history of falls exhibit a smaller difference in functional activation between cognitively different conditions, as well as an overall reduction in BOLD signal during an executive cognitive task. Generally, future work aimed at identifying underlying brain structure and function responsible for impaired cognitive processing in older adults at-risk for falls will augment our current understanding of the mechanisms underlying falls – and aid prevention and treatment efforts.

## Conflict of Interest Statement

The authors declare that the research was conducted in the absence of any commercial or financial relationships that could be construed as a potential conflict of interest.

## AUTHOR CONTRIBUTIONS

All authors contributed to study concept. In addition, Lindsay S. Nagamatsu was responsible for data collection, data analysis and interpretation, and manuscript preparation. Lara A. Boyd was responsible for data analysis and interpretation and critical review of the manuscript, Chun Liang Hsu was responsible for data collection and critical review of the manuscript. Todd C. Handy was responsible for data interpretation and critical review of the manuscript. Teresa Liu-Ambrose was responsible for data collection, data interpretation, and manuscript preparation.
